# Fumonisin B_1_ Induces Oxidative Stress and Breaks Barrier Functions in Pig Iliac Endothelium Cells

**DOI:** 10.3390/toxins11070387

**Published:** 2019-07-02

**Authors:** Qiaoling Yuan, Yancheng Jiang, Ying Fan, Yingfeng Ma, Hongyu Lei, Jianming Su

**Affiliations:** 1 Department of Basic Veterinary Medicine, College of Veterinary Medicine, Hunan Agricultural University, Changsha 410128, China; 2 Department of Preventive Veterinary Medicine, College of Veterinary Medicine, Hunan Agricultural University, Changsha 410128, China

**Keywords:** Fumonisin B_1_, pig iliac endothelial cells, oxidative stress, tight junction

## Abstract

Fumonisins (Fums) are types of mycotoxin that widely contaminante feed material crops, and can trigger potential biological toxicities to humans and various animals. However, the toxicity of Fums on porcine blood vessels has not been fully explored. Fumonisin B_1_ (FB_1_) is the main component of Fums. Therefore, the aim of this study was to explore the effects of FB_1_ on the oxidative stress and tight junctions of the pig iliac endothelial cells (PIECs) in vitro. The results showed that FB_1_ reduced the viability of PIECs, increased the contents of lipid peroxidation product malondialdehyde (MDA), decreased the activities of antioxidant enzymes superoxide dismutase (SOD), glutathione peroxidase (GSH-Px), catalase (CAT) and thioredoxin reductase (TrxR), and decreased the level of glutathione (GSH). In addition, the barrier functions were destroyed, along with the down-regulations on Claudin 1, Occludin and ZO-1 and the increase of paracellular permeability. Thus, this research indicates that FB_1_ facilitates oxidative stress and breaks barrier functions to damage pig iliac endothelium cells.

## 1. Introduction

Fumonisins (Fums) are water-soluble secondary metabolites produced by *Fusarium verticillioides* [1]. So far, many types of Fums have been discovered, including FA_1_, FA_2_, FB_1_, FB_2_, FB_3_, FB_4_, FC_1_, FC_2_, FC_3_, FC_4_ and FP_1_, etc. FB_1_ is characterized by having the highest proportion and strongest toxicity among them [2,3]. Moreover, their water-soluble property and high thermostability make FB_1_ a risk factor for humans and animals [4]. The contamination of FB_1_ in foods and feeds such as corns and corn-based foods/feeds, is very common all over the world. According to Food and Agriculture Organization of the United Nations and World Health Organization (FAO/WHO), the maximum tolerable daily intake (TDI) of Fums for humans is 2–4 mg/kg and for animals is 5–100 mg/kg [5]. For humans, the main harmful effect of Fums is the induction of esophageal cancer and neural tube defects [6,7]. However, with excessive intake of Fums, the digestive system, immune system, reproductive system, neuroendocrine system of livestock and poultry are severely damaged [8,9,10]. Pulmonary edema is a typical symptom of pigs exposed to Fums [9]. It has been shown that alterations of endothelial cells were presented in lung capillaries of pigs fed with FB_1._ [10]. FB_1_ was also demonstrated to inhibit porcine granulosa cell proliferation at the concentration of 10 µM, suggesting a negative effect of FB_1_ on ovarian function [11]. Moreover, continuous FB_1_ treatment for 3 months at 10 ppm led to an increase in vascular permeability of the lungs, brain and kidneys of piglets [12].

Oxidative stress refers a pathological process characterized by an imbalanced status of oxidation and anti-oxidation, which is known to be a major cause of endothelial dysfunction, and plays a key role in the development of vascular disease [13,14]. Oxidative stress induces vascular endothelial cell injury by over-producing oxygen free radicals, which is closely related to several disorders such as atherosclerosis and hypertension [15]. Solid evidence showed that in endothelial cells, oxidative reactions are generally evoked by oxidized low-density lipoprotein, low levels of nitric oxide and vascular inflammation [16], indicating that oxidative reactions are involved in the process of endothelial cell injury and functional changes. Furthermore, FB_1_ aggravates oxidative stress in porcine kidney cells by reducing the activities of anti-oxidative substance glutathione (GSH) [17] and elevating the content of malondialdehyde (MDA) [18]. However, there are no studies to explain the relationship between oxidative stress of porcine vessel lesion and FB_1_-induced toxicities.

Endothelial cells are the interface between circulating blood and surrounding tissues. They contact circulating blood directly. Moreover, vascular endothelial cells are the earliest affected targets by the components in blood [19,20]. Consequently, vascular endothelial barrier functions play an important role in maintaining the internal homeostasis [21]. Endothelial dysfunction is considered to be an early event leading to vascular wall disorders, including cell membrane damage and the increase of permeability, and even cell swelling and necrosis [22]. In addition, the occurrence of diseases including atherosclerosis and hypertension is closely associated with the lesion of barrier functions of vascular endothelial cells [15]. As a part of the circulatory system, blood vessels are the available target sites of harmful substances entering the blood. However, the negative effects of FB_1_ on porcine vessel remains unknown. Thus, in our study, pig iliac endothelial cells (PIECs) were selected as the object to research the impact of oxidative stress and tight junction caused by FB_1_.

## 2. Results

### 2.1. FB_1_ Suppressed the Viability of PIECs

**Figure 1:**
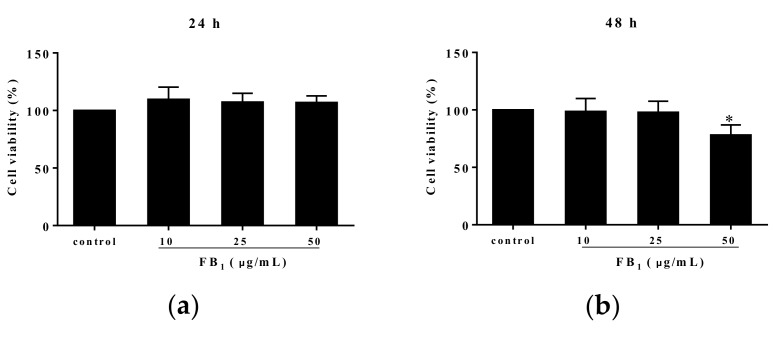

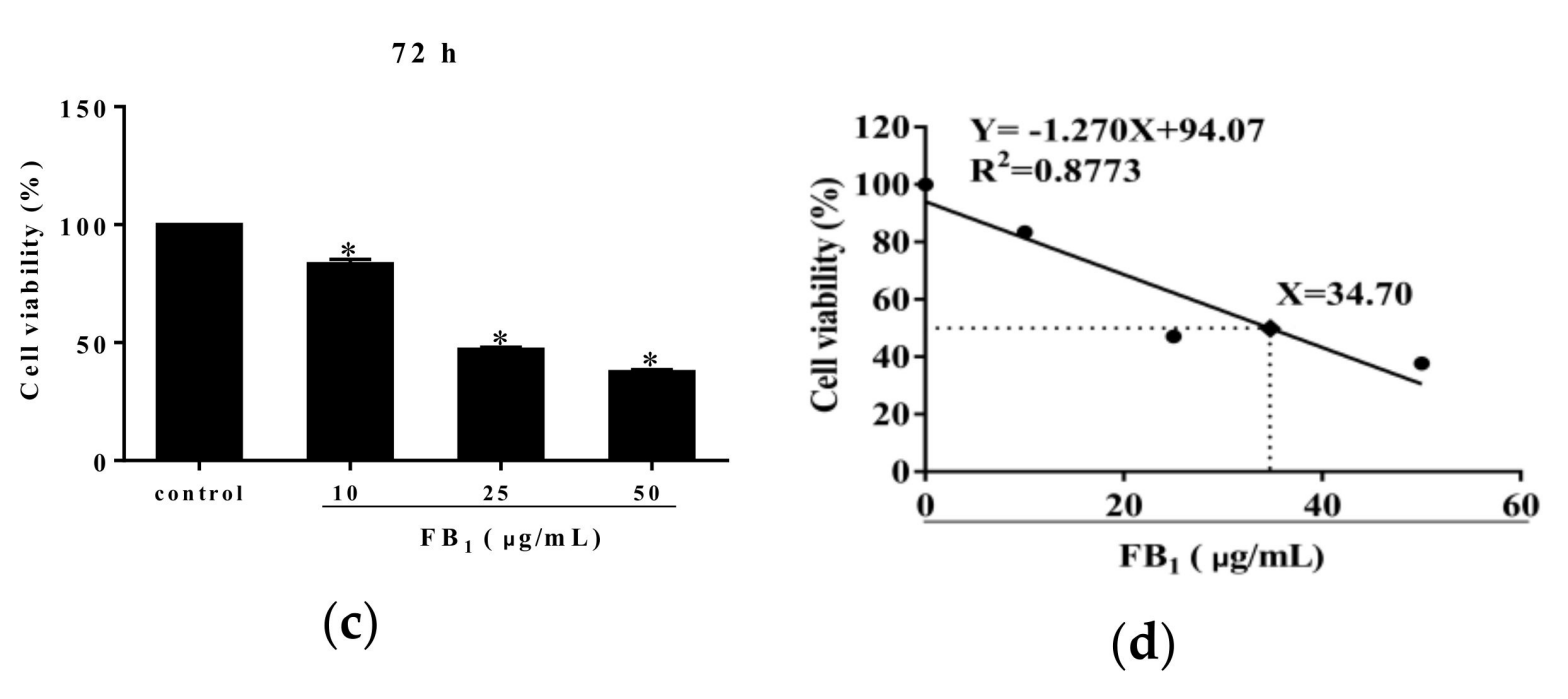
The effect of FB_1_ treatment on cell viability of PIECs. The cell viability of PIECs exposed to different concentrations of FB_1_ for 24 h (**a**); The cell viability of PIECs exposed different concentrations of FB_1_ for 48 h (**b**); The cell viability of PIECs exposed different concentrations of FB_1_ for 72 h (**c**); The cell viability curve of PIECs exposed to FB_1_ for 72 h (**d**). The values were represented as ± standard deviation (SD), * *p* < 0.05, compared with control group.

Methyl thiazolyl tetrazolium (MTT) analysis showed that FB_1_ treatment for 24 h at the various concentrations of 10, 25, and 50 µg/mL did not alter PIECs viability (*p* > 0.05) (Figure 1a). However, 50 µg/mL of FB_1_ exposure for 48 h significantly reduced cell viability, compared with control group (*p* < 0.05) (Figure 1b). Additionally, we found an obvious decline at 72 h in viability of PIECs challenged with FB_1_ at the concentrations of 10, 25, and 50 µg/mL in a concentration-dependent manner (Figure 1c). Based on a linear relationship (*Y* = −1.270*X* + 94.07, *R*^2^ = 0.8773, *Y* was cell viability, and *X* was the concentration of FB_1_) between cell viability and FB_1_ concentrations, the concentration of FB_1_ inducing 50% of cell viability was calculated, which was the median lethal concentration of FB_1_ to PIECs at 72 h was 34.70 µg/mL (Figure 1d). At 48 h, the median lethal concentration of FB_1_ to PIECs was above 50 μg/mL.

### 2.2. FB_1_ Exerted Oxidative Stress in PIECs

**Figure 2:**
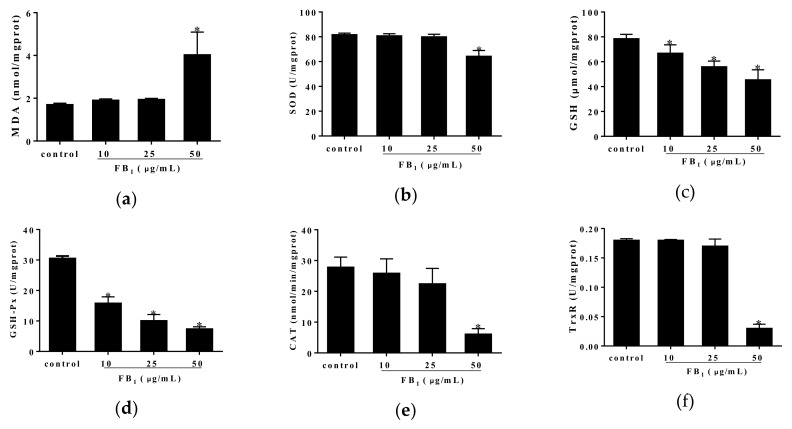
The effect of FB_1_ treatment for 48 h on oxidative stress of PIECs. The oxidation indices of MDA (**a**), SOD (**b**), GSH (**c**), GSH-Px (**d**), CAT (**e**) and TrxR (**f**). Data were represented as means ± SD, * *p* < 0.05, compared with control group.

In order to better understand the correlation between FB_1_-caused cell injury and oxidative stress, we further examined oxidative indices including MDA, superoxide dismutase (SOD), GSH, glutathione peroxidase (GSH-Px), catalase (CAT) as well as thioredoxin reductase (TrxR). We found that elevated content of MDA and decreased content of GSH were caused by FB_1_ treatment for 48 h, accompanied with evident decreases in activities of anti-oxidative enzymes such as SOD, GSH-Px, CAT and TrxR (Figure 2a–f). Accordingly, our findings suggested that the oxidative stress was tightly associated with FB_1_-induced damage in PIECs.

### 2.3. FB_1_ Exacerbated the Permeability of PIECs

**Figure 3:**
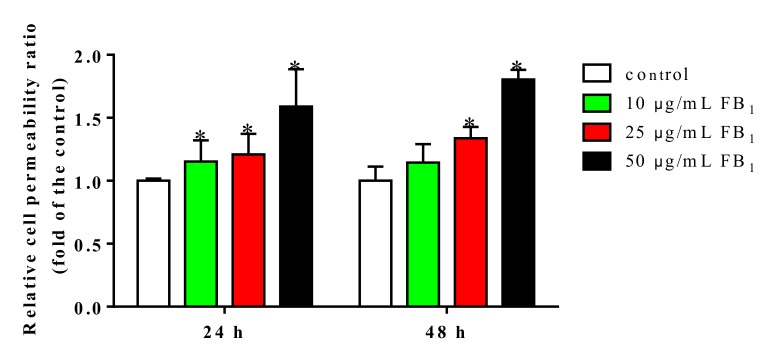
FB_1_ increased permeability of PIECs. The endothelial permeability was determined by the Transwell assay. The values were represented as means ± SD, * *p* < 0.05, compared with control group.

To further confirm the damage of FB_1_ on endothelial barrier functions, we examined the flux of fluorescein isothiocyanate-dextran (FITC-dextran) across PIECs monolayer. Untreated PIECs monolayer exhibited a normal paracellular passage. However, upon treatment with 25 and 50 µg/mL of FB_1_ for 24 and 48 h, PIECs monolayer became significantly permeable (Figure 3). Consequently, tight junctions of PIECs were damaged after FB_1_-treatment, in company with the increased paracellular permeability.

### 2.4. FB_1_ Destructed the Tight Junctions in PIECs

**Figure 4:**
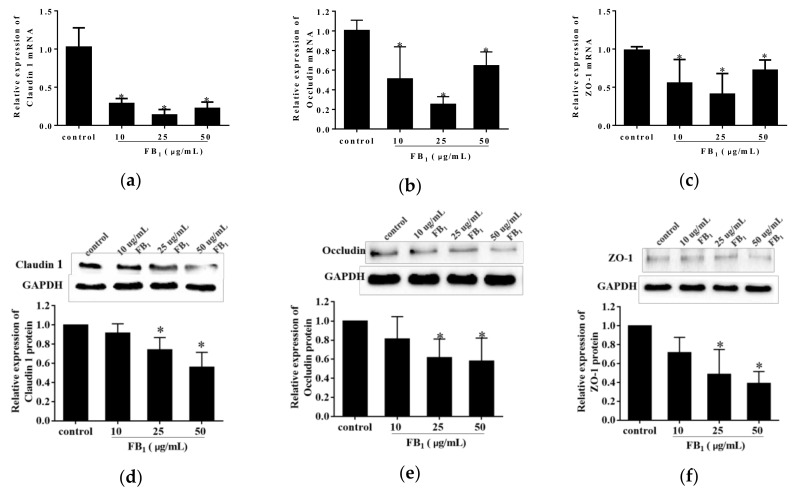
FB_1_ restrained the expressions of Claudin 1, Occludin, and ZO-1 in PIECs. The quantitative qPCR and western blot analysis of Claudin 1 (**a**,**d**), Occludin (**b**,**e**), and ZO-1 (**c**,**f**) were performed. In western blot analysis, the glyceraldehyde phosphate dehydrogenase (GAPDH) served as the internal reference control. The values were represented as means ± SD, * *p* < 0.05, compared with control group.

To further explore the action of FB_1_ on paracellular permeability, we detected the effect of FB_1_ on expression levels of tight junction proteins on mRNA level and protein level in FB_1_-treated PIECs using real-time quantitative polymerase chain reaction (qPCR) and western blot analysis. FB_1_ exposure for 48 h caused a remarkably decrease in the mRNA and protein levels of Claudin 1, Occludin and ZO-1 (*p* < 0.05) (Figure 4a–f). Therefore, our results indicated that the PIECs barrier functions were impaired after FB_1_ treatment.

## 3. Discussion

Fums are widely presented in maize, cereals and other crops as well as animal feed all over the world, which is a critical harmful factor that causes livestock diseases. For example, they can lead to horse encephalomalacia [23], pig pulmonary edema [24], and even animal reproductive disorders [25], causing tremendous economic losses to the livestock and poultry breeding industry. Although there are many ways, including the use mound inhibitors, to relieve the harm of mycotoxins on animal health, the problems arising from the contamination of mycotoxins in foods and feeds are still serious. The content of Fums (total = FB_1_ + FB_2_ + FB_3_) was 6865 µg/kg in the third season in white maize in the North West region of South Africa (2015–2016) [26]. However, so far, there have been few reports about the effects of mycotoxins on porcine vascular endothelial cells. In this experiment, we discovered that FB_1_ led to oxidative stress and barrier dysfunction in PIECs, which provides a novel insight into the toxicity of FB_1_ in porcine vessels.

Fums have a variety of cytotoxic effects and have been proven to inhibit proliferation and affect cell functions in a variety of cells of pigs. Our results indicated that FB_1_ could decrease the cell viability at the concentration of 50 µg/mL for 48 h. However, 10–50 µg/mL of FB_1_ exposure for 72 h significantly reduced cell vitality. These results suggest that FB_1_ inhibits PIECs viability in a time-and dose-dependent manner. Similarly, the cell viability of porcine pulmonary artery endothelial was decreased significantly by 25 μM of FB_1_ treatment for 24 h [27]. Previous studies also showed that 40 µg/mL of FB_1_ could inhibit cell viability of porcine jejunal epithelial cells [28]. It was also found that the viability of porcine renal epithelial cells was restrained by 10 µM of FB_1_ [29].

The destruction of intracellular redox balance has been identified as one of the key underlying factors for animal diseases. Overproduction of reactive oxygen species is the main property of oxidative damage, and MDA is the final product of lipid peroxidation of cell membrane by oxygen free radicals [30]. It is considered to be an important indicator of free radical attack. In this study, it was found that 50 µg/mL of FB_1_ treatment for 48 h increased lipid peroxidation in the PIECs. This result suggested that FB_1_ promoted cell membrane damage. SOD is an important antioxidant enzyme, which can eliminate excessive oxygen free radicals by catalytic conversion of hydroxyl radicals into hydrogen peroxide, which is then decomposed by CAT into non-toxic oxygen and water [31]. Hydrogen peroxide is a common oxygen free radical and participates in impairment of cells or tissues. We found that FB_1_ reduced the activities of SOD and CAT in cells, thus depressing the resistance of cells to oxidative damage. Previous studies showed that Fums decreased the activity of CAT of the liver and kidney in mice [32]. Besides, GSH is also a free radical scavenger and considered as an early biomarker of oxidative stress [33]. GSH and GSH-Px play an irreplaceable role in maintaining intracellular redox environment and protecting cells from oxidative damage. GSH can interact directly with reactive oxygen species and is a catalytic substrate of GSH-Px [34]. Decreased levels of GSH and GSH-Px further demonstrated that FB_1_ caused oxidative damage to PIECs. Synchronously, TrxR, one of the key enzymes in GSH redox cycle, was also decreased, which further indicated that FB_1_ facilitated the oxidative stress. It has been shown that the redox system is attacked in vivo and in vitro when the cell and tissues are exposed to FB_1_. The main characteristics of oxidative damage are the increase in the levels of ROS and MDA, and the decrease in the levels of antioxidant GSH, GPX and SOD [35]. A previous study has found that FB_1_ not only increased MDA content of porcine kidney cells PK15 at the concentration of 10 µM, but also decreased GSH content of PK15 at 0.05–5 µg/mL [18,36]. It was found that the content of MDA and the activity of SOD increased, and the activity of GSH decreased in the spleens of mice that ingested 100 µg/kg of FB_1_ daily [37]. FB_1_ could induce oxidative stress in hepatocellular carcinoma cell line HepG2 by the increased content of MDA, and the decreased activities of CAT, SOD [38,39]. The activity of CAT, SOD and TrxR were decreased in the livers of mice after FB_1_ treatment at the dosage of 2.5 mg/kg [38].

Endothelial cell junction includes tight junction and adhesion junction. Tight junction participates in maintaining the integrity of endothelial cell barrier functions [40]. Claudins, Occludin and ZO-1 are common tight junction proteins, which play important roles in maintaining cell morphology and forming barriers to prevent the invasion of pathogens [41]. The paracellular permeability of PIECs was increased by FB_1_ exposure at 10 and 50 µM FB_1_ at 50 µM destroyed the cell barrier functions of porcine pulmonary artery endothelial cells and even caused cell death [27]. The intestinal transcellular and paracellular permeabilities of piglets were increased by Fums exposure [42]. Meanwhile, we demonstrated that the expression levels of Claudin-1, Occludin and ZO-1 was reduced by FB_1_ treatment for 48 h. Similarly, FB_1_ altered the barrier function of intestinal cells for for a long time [43]. These results suggested that mycotoxins FB_1_ affected the expressions of tight junction proteins in porcine vessel endothelial cells and might alter cell-to-cell connectivity.

## 4. Conclusions

All in all, 48 h treatment of FB_1_ exerts impairment in PIECs via inducing oxidative stress and barrier destruction. These findings further provide a novel insight to the specific toxicity of FB_1_ in porcine vessels and provide a useful basis for research into reducing its harms to pigs.

## 5. Materials and Methods

### 5.1. Cell Line and Cell Culture

PIECs were purchased from the Cell Bank of Shanghai Academy of Chinese Sciences. Cells were cultured in Roswell Park Memorial Institute (RPMI) medium 1640 (Gibco, Shanghai, China), with 10% of fetal bovine serum (FBS) (Zhejiang Tianhang Biotechnology Co., Ltd., Hangzhou, China) at the condition of 37 °C and 5% CO_2_.

### 5.2. Cell Viability Assay by MTT

Cell viability analysis was determined by MTT assay. The FB_1_ (≥ 98%, Sigma-Aldrich, St. Louis, MO, USA and Alfa Aesar, Haverhill, MA, USA) was dissolved in dimethyl sulfoxide (DMSO) (Solarbio Biotechnology Co., Ltd. Shanghai, China) solution to prepare FB_1_ stock solution. FB_1_ stock solution was diluted into the working solution at the concentration of 0, 10, 25 and 50 µg/mL by fresh basic medium. The group of 0 µg/mL of FB_1_ treatment was regarded as the control group. Cells were inoculated into 96-well culture plates at 2 × l0^3^/well. The cells were incubated for 24 h then were treated with 0, 10, 25, and 50 µg/mL FB_1_ for 24, 48 or 72 h. Following this, 10 µL MTT (Beyotime Biotechnology, Shanghai, China) reagent with 100 µL culture medium was added into each well. After 4 h, 150 µL of formazan solvent DMSO was added. The plates were shaken slightly for 10 min, followed by absorbance measuring at 490 nm with a microplate reader. Calculating formula of cell viability was followed: Cell viability (%) = (OD_treated_ − OD_blank_)/(OD_control_ − OD_blank_) × 100%. Based on a linear relationship between cell viability and FB_1_ concentrations, the concentration of FB_1_ inducing 50% of cell viability was calculated.

### 5.3. Detection of Oxidation Indices

The cells were plated at a density of 1 × 10^5^ /well pre-culture for 24 h. Next, cells were incubated with different concentrations of FB_1_ for 48 h. The contents of MDA and GSH and the activities of SOD, GSH-Px and TrxR were detected according to the operation requirements of corresponding kits (NanJing JianCheng Bioengineering Institute, Nanjing, China). Additionally, the activity of CAT was determined by the biochemical assay kit (Suzhou Keming Biotechnology, Suzhou, China). The calculation method of each indicator was based on each detection kit.

### 5.4. Measurement of Paracellular Permeability

Paracellular permeability was detected by FITC-dextran (Sigma-Aldrich, St. Louis, MO, USA and Alfa Aesar, Haverhill, MA, USA). PIECs were inoculated into the upper chamber. After filling the upper chambers, the cells were treated with different concentrations of FB_1_ for 24 h and 48 h respectively. Then FITC-dextran solution was added to the upper chamber with a final concentration of 5 mg/mL for 3 h treatment. Finally, the concentration of the FITC-dextran in the lower chamber was determined at excitation wavelengths of 480 nm and emission wavelengths of 520 nm using a fluorometer. According to the established standard curve of FITC-dextran, the concentration of FITC-dextran in the lower chamber was calculated. Calculating formula of relative cell permeability ratio was as followed: Relative cell permeability (flod) = Concentration_treated_/Concentration_control_.

### 5.5. qPCR Assay

The cells were incubated with FB_1_ of 0, 10, 25, and 50 µg/mL for 48 h after pre-cultured for 24 h in 96-wells. The total RNA of cells was extracted by TransZol UP kit (TransGen Biotech, Beijing, China). The reverse transcription was carried out according to the instructions of one-step reverse transcription kit (TransGen Biotech, Beijing, China). The produced cDNA was diluted to 100 ng/mL, and the subsequent qPCR was conducted by the commercial kit (Vazyme Biotech, Nanjing, China). Gene relative expression levels were calculated using the 2^(−ΔΔCT)^ method. The house-keeping gene was GADPH. The primers used in this study were synthesized by Sangon Biotech Co., Ltd. (Shanghai, China) (Table 1). 

### 5.6. Western Blot

After a pre-incubation, PIECs were incubated with different concentrations of FB_1_ for 48 h. Extraction of total protein of cells in each group was carried out strictly according to the instructions using Radio Immunoprecipitation Assay (RIPA) (Solarbio Biotechnology Co., Ltd. Shanghai, China) lysate at 4 °C. The protein concentrations were determined by Bradford method for adjusting sampling mass. After separation by polyacrylamide gel electrophoresis, the proteins were transferred to the polyvinylidene fluoride membranes, and the membranes were sealed at room temperature for 1 h, and then were incubated with primary antibodies: ZO-1, Occludin, Claudin 1, and GAPDH (Boiss Biotechnology Co., Ltd., Beijing, China) at 4 °C, overnight, followed by incubation with the secondary antibody at room temperature for 1 h. The grey level of each protein was detected by the Electro-Chemi-Luminescence (ECL) detection kit (Jiangsu KeyGEN BioTech, Nanjing, China).

### 5.7. Statistical Analysis

All data were expressed as mean ± SD. The data were analyzed by the single factor analysis and independent sample *t* test using GraphPad Prism 6.01 (GraphPad Software, La Jolla, USA, 2010). All tests were repeated three or more times. A *p* more than 0.05 indicated no significant difference; and *p* lower than 0.05 indicated significant difference.

## Figures and Tables

**Table 1:** Primer sequences of genes for qPCR.

Gene Name	Sequences	Genebank No.
Claudin 1	F: 5′- GCAGCAGCTTCTTGCTTCTC-3′	NM_001244539.1
	R: 5′-CTGGCATTGACTGGGGTCAT-3′	
Occludin	F: 5′- ATCAACAAAGGCAACTCT-3′	XM_005672525.3
	R: 5′-GCAGCAGCCATGTACTCT-3′	
ZO-1	F: 5′- GAGTTTGATAGTGGCGTT-3′	XM_021098896.1
	R: 5′- GTGGGAGGATGCTGTTGT-3′	
GAPDH	F: 5′- ACAGGGTGGTGGACCTCATG-3′	XM_021091114.1
	R: 5′-GGGTCTGGGATGGAAACTGG-3′	
